# Response to “Incidence of hypotension according to the discontinuation order of vasopressors: a matter of pharmacokinetics”

**DOI:** 10.1186/s13054-018-2166-y

**Published:** 2019-04-24

**Authors:** Kyeongman Jeon, Jae-Uk Song, Gee Young Suh

**Affiliations:** 1 Department of Critical Care Medicine, Samsung Medical Center, Sungkyunkwan University School of Medicine, Seoul, Republic of Korea; 2 Division of Pulmonary and Critical Care Medicine, Department of Medicine and, Samsung Medical Center, Sungkyunkwan University School of Medicine, 81 Irwon-ro, Gangnam-gu, Seoul, 06351 Republic of Korea; 3 Division of Pulmonary and Critical Care Medicine, Department of Medicine, Kangbuk Samsung Hospital, Sungkyunkwan University School of Medicine, Seoul, Republic of Korea

We thank Drs Freebairn and Hollander for their thoughtful comments [1] regarding our recent randomized controlled trial on the incidence of hypotension while tapering vasopressors in patients on concomitant norepinephrine and vasopressin recovering from septic shock [2]. We understand their concern that the difference in half-lives of two vasopressors could influence the hemodynamic tolerance to the vasoactive drug tapering. The longer effective half-life of vasopressin compared with norepinephrine may help avoid rebound hypotension during tapering of the drug [3]. Drs Freebairn and Hollander also suggest that the interval of vasopressor tapering should have been longer for vasopressin compared with norepinephrine due to different half-lives of the drugs. The fact that the median time to hypotension after vasopressor tapering was shorter in the norepinephrine-tapered first group than the vasopressin-tapered first group might be considered to support this assumption. First of all, we want to point out that both vasopressors were tapered by one-third of the original dosage every hour, respectively, thus making the serum level more comparable than that assumed by Drs Freebairn and Hollander in their letter [1]. Also, the time to hypotension is the time from the start of vasopressor tapering to the appearance of hypotension, not the time from the last reduction of vasopressor dosage to hypotension. Therefore, we reanalyzed the data to look at the time to appearance of hypotension from the time of latest reduction in vasopressor dosage. In those experiencing hypotension, hypotension occurred at 23 (7–34) min after the latest dose reduction of norepinephrine dose compared with at 24 (13–54) min after the latest reduction in vasopressin dose, which was not statistically significant (*P* = 0.288). Therefore, we think it is highly unlikely that our observed finding of increased incidence of hypotension during norepinephrine tapering was related to differences in the half-lives of the two vasopressors. In addition, 1 hour of observation period should be enough for assessing hypotension that developed when tapering vasopressin infusion.
